# IL 33 Correlates With COVID-19 Severity, Radiographic and Clinical Finding

**DOI:** 10.3389/fmed.2021.749569

**Published:** 2021-11-30

**Authors:** Sofija Sekulic Markovic, Marina Jovanovic, Nevena Gajovic, Milena Jurisevic, Nebojsa Arsenijevic, Marina Jovanovic, Milan Jovanovic, Zeljko Mijailovic, Snezana Lukic, Nenad Zornic, Vladimir Vukicevic, Jasmina Stojanovic, Veljko Maric, Miodrag Jocic, Ivan Jovanovic

**Affiliations:** ^1^Department of Infectious Disease, Faculty of Medical Sciences, University of Kragujevac, Kragujevac, Serbia; ^2^Faculty of Medical Sciences, Center for Molecular Medicine and Stem Cell Research, University of Kragujevac, Kragujevac, Serbia; ^3^Department of Clinical Pharmacy, Faculty of Medical Sciences, University of Kragujevac, Kragujevac, Serbia; ^4^Department of Virusology and Immunology, Institute for Public Health Kragujevac, Kragujevac, Serbia; ^5^Department of Internal Medicine, Faculty of Medical Sciences, University of Kragujevac, Kragujevac, Serbia; ^6^Department of Abdominal Surgery, Military Medical Academy, Belgrade, Serbia; ^7^Department of Radiology, Faculty of Medical Sciences, University of Kragujevac, Kragujevac, Serbia; ^8^Department of Surgery, Faculty of Medical Sciences, University of Kragujevac, Kragujevac, Serbia; ^9^University Clinical Center Kragujevac, Kragujevac, Serbia; ^10^Department of Otorhinolaringology, Faculty of Medical Sciences, University of Kragujevac, Kragujevac, Serbia; ^11^Department of Surgery, Faculty of Medicine Foca, University of East Sarajevo, Foca, Bosnia and Herzegovina; ^12^Institute for Transfusiology and Haemobiology, Military Medical Academy, Belgrade, Serbia

**Keywords:** IL 33, COVID-19, disease severity, correlation, proinflammatory innate immune response

## Abstract

**Objective:** The increased level of interleukin (IL)-33 is considered as a predictor of severe coronavirus disease 2019 (COVID-19) infection, but its role at different stages of the disease is still unclear. Our goal was to analyze the correlation of IL-33 and other innate immunity cytokines with disease severity.

**Methods:** In this study, 220 patients with COVID-19 were included and divided into two groups, mild/moderate and severe/critical. The value of the cytokines, clinical, biochemical, radiographic data was collected and their correlation with disease severity was analyzed.

**Results:** Most patients in the severe/critical group were male (81.8%) and older (over 64.5 years). We found a statistically significant difference (*p* < 0.05) in these two groups between clinical features (dyspnea, dry cough, fatigue, and auscultatory findings); laboratory [(neutrophil count, lymphocyte count, monocyte count, hemoglobin, plasma glucose, urea, creatinine, total bilirubin (TBIL), direct bilirubin (DBIL), aspartate aminotransferase (AST), albumin (ALB), lactate dehydrogenase (LDH), creatinine kinase (CK), D-dimer, C-reactive protein (CRP), procalcitonin (PCT), Fe, and Ferritin)], arterial blood gases (oxygen saturation-Sa0_2_, partial pressure of oxygen -p0_2_), and chest X-rays (CXR) lung findings (*p* = *0*.000). We found a significantly higher serum concentration (*p* < 0.05) of TNF-α, IL-1β, IL-6, IL-12, IL-23, and IL-33 in patients with COVID-19 with severe disease. In the milder stage of COVID-19, a positive correlation was detected between IL-33 and IL-1β, IL-12 and IL-23, while a stronger positive correlation between the serum values of IL-33 and TNF-α, IL-1β, IL-6, and IL-12 and IL-23 was detected in patients with COVID-19 with severe disease. A weak negative correlation (*p* < 0.05) between pO_2_ and serum IL-1β, IL-12, and IL-33 and between SaO_2_ and serum IL-33 was noted. The positive relation (*p* < 0.05) between the serum values of IL-33 and IL-12, IL-33 and IL-6, and IL-6 and IL-12 is proven.

**Conclusion:** In a more progressive stage of COVID-19, increased IL-33 facilitates lung inflammation by inducing the production of various innate proinflammatory cytokines (IL-1β, IL-6, TNF-α, IL-12, and IL-23) in several target cells leading to the most severe forms of the disease. IL-33 correlates with clinical parameters of COVID-19 and might represent a promising marker as well as a therapeutic target in COVID-19.

## Introduction

In December 2019, in Wuhan, Hubei province, China, several patients with atypical pneumonia were hospitalized (1). A new virus from the group of coronaviruses was identified and called severe acute respiratory syndrome coronavirus 2 (SARS-CoV-2) and the disease called Coronavirus Disease (COVID-19) (2). SARS-CoV-2 is highly infectious and asymptomatic patients are the main source of infection (3). Because of its easy and quick transmission and interhuman spread, the WHO declared it as a pandemic on March 11, 2020 (1). Recent studies have shown that different biochemical parameters are altered in patients with COVID-19, and some of them are useful as indicators of disease severity (4–6). Patients can have various forms of the disease, from asymptomatic to acute respiratory distress syndrome (ARDS) and respiratory insufficiency (7, 8). The most common manifestations are fever, cough, dyspnea, fatigue, chest pain, loss of sense of smell, headache, nausea or vomiting, and pharyngalgia (7, 9). Chest X-rays (CXR) is not considered a sensitive method for the detection of lung abnormalities in the early stage of the disease, but it is useful for monitoring the lung progression in later stages of the disease (10, 11). Immunopathogenic mechanisms during the disease vary from immunosuppression to uncontrolled immune-inflammatory response (12, 13). While the immunosuppression is conveyed through well-known immunosuppressive cytokines such as transforming growth factor β (TGF-β) and interleukin (IL)-10, the uncontrolled immune response is manifested by the rapid production and excretion of proinflammatory cytokines, such as cytokines of the IL-1 family, tumor necrosis factor α (TNFα), and IL-6. (12, 14) The rapid secretion of cytokines, chemokines, and other proinflammatory molecules, often referred to as cytokine storm, can be clinically manifested as ARDS with consequent respiratory failure (15, 16). Even though ARDS and cytokine storm are well-established entities in modern medicine, the exact mechanisms of respiratory failure and impeding proinflammatory cascade in COVID-19 are still challenging but might be of great significance when it comes to predicting the outcome of the disease and treatment (17).

One of the cytokines that have not been sufficiently studied in the immunopathogenesis of COVID-19 is IL-33. As a member of the IL-1 family, IL-33 induces an inflammatory response within the tissue (18–20). In the lungs, it is secreted mainly by injured epithelial alveolar cells (10, 19). As a previous study showed that the virus can trigger the secretion of IL-33 through activation of SARS CoV-2-derived papain-like protease (PLpro), IL-33 is considered as a predictor of severe forms of COVID-19. However, the potential role of IL-33 in disease genesis and progression has not been studied sufficiently (18, 19).

In this study, we analyzed the correlation of IL-33 and other innate immunity cytokines such as TNF-α, IL-1β, IL-6, IL-12, and IL-23 with disease severity. Also, we analyzed interdependence between disease severity and clinical, biochemical features, and CXR lung findings in 220 patients with COVID-19.

## Methods

### Patients

In this observational and cross-sectional study, confirmed cases of COVID-19 from May 2020 to December 2020 were involved and examined. COVID-19 was confirmed by real-time reverse transcription–PCR (RT-PCR) analysis. All hospitalized patients with COVID-19 were classified according to disease severity into two groups:

I) Mild/moderate group, patients with fever (37–38°C), fatigue, pharyngalgia, anosmia, nausea and vomiting, headache, dry irritating cough, dyspnea, SaO_2_ 82–100%, pO_2_ 7.1 kPa- 13.3 kPa, with normal or weakened/sharpened respiratory noise, and audible cracks in the lower segments of the lungs, with normal/interstitial thickening and focal consolidation in CXR lung findings (CXR I, II, III);
II) Severe/critical group, patients with fever of 38–40°C, frequent dry irritating cough, dyspnea, fatigue, myalgia, nausea and vomiting, headache, chest pain, anosmia, SaO2 ≤ 70–81%, and pO2 ≤ 5.1–7 kPa which require high-frequency ventilation (HFV), non-invasive mechanical ventilation (Non-Invasive Ventilation, NIV), or invasive mechanical ventilation, with auscultatory attenuated-inaudible respiratory noise with audible whistling or cracks diffusely, with multifocal consolidation, diffuse alveolar changes, ARDS in CXR lung findings (CRX IV, V).

The patients included in this study had no prior treatment with antibiotics, aminosalicylates, corticosteroids, statins, immunosuppressive agents, or any kind of biological therapy for at least 2 months before enrolling in the study.

A total of 220, 110 patients from each of the previously defined groups, were selected at random for the study. The study sample of 110 patients from each group was extracted by simple randomization technique, activating for 110 times random number generator in Excel (RANDBETWEEN).

### Data Collection

Nasopharyngeal swab samples were taken from all patients with suspected SARS-CoV-2 infection and then sent to designated authoritative laboratories to detect the SARS-CoV-2. For the detection and quantification of viral nucleic acid, we used the RT-PCR for the N gene and Orf gene of SARS-CoV-2. Reverse transcription, duplication, and detection are performed automatically in a JENA Real-time Thermal Cycler. We used specific primers and a fluorescence probe. Samples were considered as positive if an amplification signal was detected on the detection system and internal control, and negative in the absence of any amplification signal on the detection system and a positive result on internal control. The assay was repeated in the absence of signal in the positive control and the presence of a negative control amplification signal.

Blood samples were drawn by venous puncture from all patients included in the study, and stored in four different tubes: one for blood cell counting, another tube for D-dimer, the third one for analyses of the biochemistry in plasma [urea, glycemia, creatinine, C-reactive protein (CRP), procalcitonin (PCT), creatinine kinase (CK), aspartate aminotransferase (AST), alanine aminotransferase (ALT), lactate dehydrogenase (LDH), albumin (ALB), direct bilirubin (DBIL), total bilirubin (TBIL), Iron (Fe), Ferritin, Potassium (K^+^), and Sodium (Na^+^)], which are performed in the Central Biochemical Laboratory of UKC Kragujevac by standard methods, using the Beckman Coulter AU 400 Unicel DXC 800 Synchron Clinical System, and fourth for immunoassay.

Several times during the day by ion selective electrode on the automaton, arterial blood gases (SaO_2_, pO_2_, partial pressure of carbon dioxide-pCO_2_), of all patients were measured.

All patients had a CXR using the digital portable anteroposterior (AP) technique. Radiographic features were described according to Yoon et al. (21) following a 5-point scoring scale: 1–normal; 2–patchy atelectasis and/or hyperinflation and/or bronchial wall thickening; 3–focal consolidation; 4–multifocal consolidation; and 5–diffuse alveolar changes.

### Measurement of Cytokine Levels in Sera

As described in our previous study, at 8:00 am, the blood was collected from the patients with COVID-19 (22). The sera were separated and stored at −80°C before use. The commercially available ELISA tests were used following the instructions of the manufacturer (R&D Systems, Minneapolis, Minn, USA) to measure the concentrations of TNF-α, IL-1β, IL-6, IL-12, IL-23, and IL-33 in serum samples.

### Statistical Analysis

Statistical Analysis Software, IBM SPSS (version 23.0) (IBM, Armonk, New York, USA) was used for performing all data analyses. The significance tests used for suitable purposes were the Chi-square test, Student's *t*-test, or Mann–Whitney U test. The data were shown as mean ± SE of the mean. Pearson's or Spearman's correlation assessed the possible relationship between variables. The strength of correlation was defined as negative or positive weak (−0.3 to −0.1 or.1 to.3), moderate (−0.5 to −0.3 or.3 to.5), or strong (−1.0 to −0.5 or 1.0 to.5). Also, the basic regression model was used to identify possible predictors for disease severity. Multiple linear regression analysis was used to predict Il-33 levels based on COVID-19 severity and other cytokine levels. Receiver operating characteristic (ROC) curve analysis was used to identify optimal cut-off values of IL33 levels in order to identify the detection of COVID-19 severity. The statistical significance was set at *p* < 0.05.

## Results

### Clinical Feature in Patients With COVID

All recruited patients met the criteria for COVID-19 (23). Among them, 140 (63.3%) were male. The mean age was 55.5 years. The COVID-19 patients were divided into two groups, based on disease severity. The patients in group II were significantly older (*p* = 0.001; Table 1). The median age in group I (mild/moderate) was 57.2 ± 1.5 and for group II (severe/critical) 64.5 ± 1.2 years. The analysis of the data did not show any significant difference regarding the distribution of gender. The analysis revealed a lower percentage of female patients and a higher percentage of male patients in group II, but this difference did not reach statistical significance (*p* = 0.071; Table 1). The most common symptoms in these two groups were fever 185 (84.0%), dry cough 176 (80%), fatigue 171 (77.7%), dyspnea 108 (49.0%), nausea and vomiting 88 (40%), and myalgia 57 (25.9%). Less common were anosmia 31 (14%), headache 29 (13.1%), chest pain 25 (11.3%), and pharyngalgia 6 (2.7%) (Table 1). The most frequent auscultatory finding in group I was attenuated breathing sound in 66.3% of patients, which CXR findings described as focal consolidation in 51.8 % and interstitial thickening in 38.1%, while in group II, 71.8% of patients had auscultatory attenuated breathing sound and 70.9% audible cracks diffusely with multifocal consolidation in 53.6% and ARDS in 46.3% of hospitalized patients. The percentage of patients with dyspnea, fatigue, auscultatory attenuated breathing sound, and audible cracks diffusely was significantly higher in the severe/critical group than in the mild/moderate group (*p* < 0.05; Table 1), which is in line with the CXR findings of the patients. The patients in the mild/moderate group had normal CRX findings, interstitial thickening, or focal consolidation zones (CRX I, II, III). Only one patient had multifocal consolidation (Table 1). All the patients in the severe/critical group had multifocal consolidation zones or diffuse alveolar changes (CRX IV, V) (*p* = 0.000). We found some significant differences in laboratory features between the mild/moderate group and the severe/critical group. In patients with severe/critical outcomes an increase in neutrophil count (*p* = 0.001), levels of plasma glucose (*p* = 0.001), urea (*p* = 0.001), creatinine (*p* = 0.001), AST (*p* = 0.002), TBIL (*p* = 0.002), DBIL (*p* = 0.017), LDH (p = 0.001), CK (*p* = 0.002), D-dimer (*p* = 0.001), CRP (*p* = 0.001), PCT (*p* = 0.001), Fe (*p* = 0.001), and Ferritin (*p* = 0.001) was noted with reduced lymphocyte count (*p* = 0.001), monocyte count (*p* = 0.006), decreased levels of hemoglobin (*p* = 0.028), and albumin (*p* = 0.001) and lower values of arterial blood gases SaO_2_ (*p* = 0.001) and pO_2_ (*p* = 0.001) compared with the mild/moderate group (Table 2). There were no statistically significant differences in white blood cell count, erythrocytes, and platelets; ALT level, K^+^, Na^+^, pH of blood, and pCO_2_ (Table 2).

**Table 1:** Demographics and clinical characteristics of patients with coronavirus disease 2019 (COVID-19).

	**Total (*n =* 220)**	**Mild/moderate (*n =* 110)**	**Severe/Critiacal (*n =* 110)**	*p* value^*^
**Age mean±SE**		57.2 ± 1.5	64.5 ± 1.27	0.001
**Sex**				
Female	80 (36.36%)	60 (54.54%)	20 (18.18%)	0.071
Male	140 (63.3%)	50 (45.5%)	90 (81.8%)	0.071
**Clinical manifestations**				
Fever	185 (84.0%)	90 (81.8%)	95 (86.36%)	1
Dry cough	176 (80%)	88 (59.9%)	88 (71%)	0.075
Fatigue	171 (77.7%)	82 (56.6%)	89 (73%)	0.008
Dyspnea	108 (49.0%)	47 (32.4%)	99 (90.0%)	0.008
Nausea and vomiting	88 (40%)	41 (37.2%)	47 (42.7%)	0.360
Myalgia	57 (25.9%)	22 (31.8%)	35 (20%)	0.271
Anosmia	31 (14.0%)	15 (13.6%)	16 (14.5%)	0.614
Headache	29 (13.1%)	14 (12.7%)	15 (13.6%)	0.627
Chest pain	25 (11.3%)	9 (8.1%)	16 (14.5%)	0.087
Pharyngalgia	6 (2.7%)	2 (1.81%)	4 (3.6%)	0.838
**Ausculattory findings**				
Normal	34 (15.4%)	27 (24.5%)	7 (6.36%)	0.003
Attenuated breathing sound	152 (69%)	73 (66.3%)	79 (71.8%)	0.032
Sharpened respiratory sound	26 (11.8%)	15 (13.6%)	11 (10%)	0.870
Audible cracks diffusely	140 (63.6%)	62 (56.3%)	78 (70.9%)	0.001
Audible whistling	5 (2.2%)	2 (1.8%)	3 (2.7%)	0.848
**CXR findings**				
Normal finding	10 (4.5%)	10 (9.0%)	0 (0%)	0.000
Interstitial thickening	42 (19.0%)	42 (38.1%)	0 (0%)	0.000
Focal consolidation	57 (25.9%)	57 (51.8%)	0 (0%)	0.000
Multifocal consolidation	60 (27.2%)	1 (0.9%)	59 (53.6%)	0.000
ARDS	51 (23.1%)	0 (0%)	51 (46.36%)	0.000

*Data expressed as mean, standard error (SR), frequency (percentage)*.

* *P values indicate differences between mild/moderate and severe/critical*.

*P < 0.05 was considered statistically significant*.

**Table 2:** Laboratory findings of patients with COVID-19.

**Measured parameters**	**Normal range**	**Mild/moderate**	**Severe/critical**	*p* value^*^
**Blood routine examination**				
White blood cell count, ×10^9^/L	3.7–10.0	7.4	8.5	
Neutrophil count %	44.0–72.0	72.7	79.1	0.001
Lymphocyte count %	20.0–46.0	17.4	12.4	0.001
Monocyte count %	2.0–12.0	0.58	0.47	0.006
Eritrocite count 10^12^/l	4.34–5.72	4.5	4.4	
Trombocite count 10^9^/l	135–450	236.2	218.2	
Hemoglobin g/L	138–175	133.3	129.2	0.028
**Biochemical examination**
Glucose mmol/L	3.8–6.1	7.1	8.4	0.001
Urea mmol/L	3.0–8.0	6.6	9.8	0.001
Creatinine umol/L	49–106	93.0	117.9	0.001
BILTumol/L	0.0–21.0	10.5	11.5	0.002
BILD umol/L	0.0–6.6	2.8	3.8	0.017
AST U/L	0–40	45.8	58.2	0.002
ALT U/L	0–40	50.1	56.9	
Albumin g/L	35–52	36.1	33.3	0.001
LDH U/L	220–450	566.0	865.0	0.001
CK U/L	0–171	191.9	313.6	0.002
D dimer ug/ml	<0.50	1.3	2.6	0.001
CRP mg/L	0.0–5.0	87.6	134.7	0.001
PCT ng/mL	0.5–2.0	0.2	0.6	0.001
K^+^ mmol/L	3.5–4.5	3.9	3.8	
Na^+^ mmol/L	136–145	136.8	136.2	
Fe umol/L	6.6–26	6.5	7.2	0.001
Ferritin ug/L	20–300	706.8	1,138.6	0.001
**Arterial blood gases**
pO_2_ kPa	10.7–13.3	9.07	6.6	0.001
pCO_2_ kPa	4.7–6.0	4.6	4.6	
SaO_2_ %	95–98	93.7	80.6	0.001
ph	7.35–7.45	7.47	7.46	

* *P values indicate differences between mild/moderate and severe/critical. P < 0.05 was considered statistically significant*.

### Patients With Severe COVID-19 Had Increased IL-33 and Proinflammatory Cytokines in Sera

**Figure 1:**
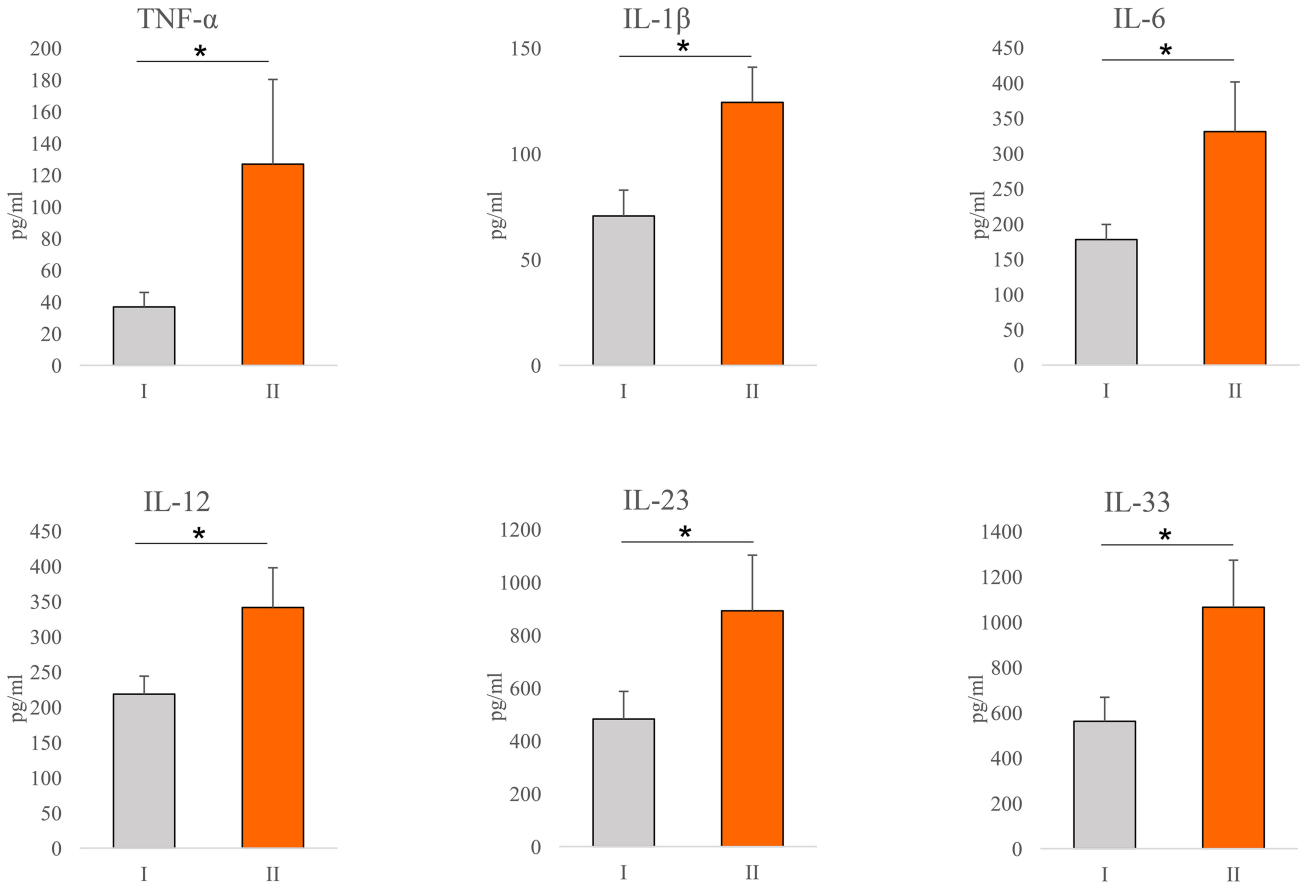
Systemic profile of innate immunity cytokines. According to the disease severity, all Coronavirus disease 2019 (COVID-19) patients were divided into two groups (I and II). The serum concentration of tumor necrosis factor α (TNFα), interleukin (IL)-1β, IL-6, IL-12, IL-23, and IL-33 were determined by ELISA. For statistical significance determination, Mann–Whitney Rank Sum test was used. *P < 0.05.

Values of cytokines of interest were analyzed in the serum of COVID-19 patients. Significantly higher concentration of TNF-α (*p* = 0.001), IL-1β (*p* = 0.001), IL-6 (*p* = 0.001), IL-12 (*p* = 0.001), IL-23 (*p* = 0.043), and IL-33 (*p* = 0.001) have been found in the sera of COVID-19 patients with severe disease (Figure 1).

### Stronger Inter-correlation Between IL-33 and Proinflammatory Cytokines in the Severe Stage of COVID-19

**Figure 2:**
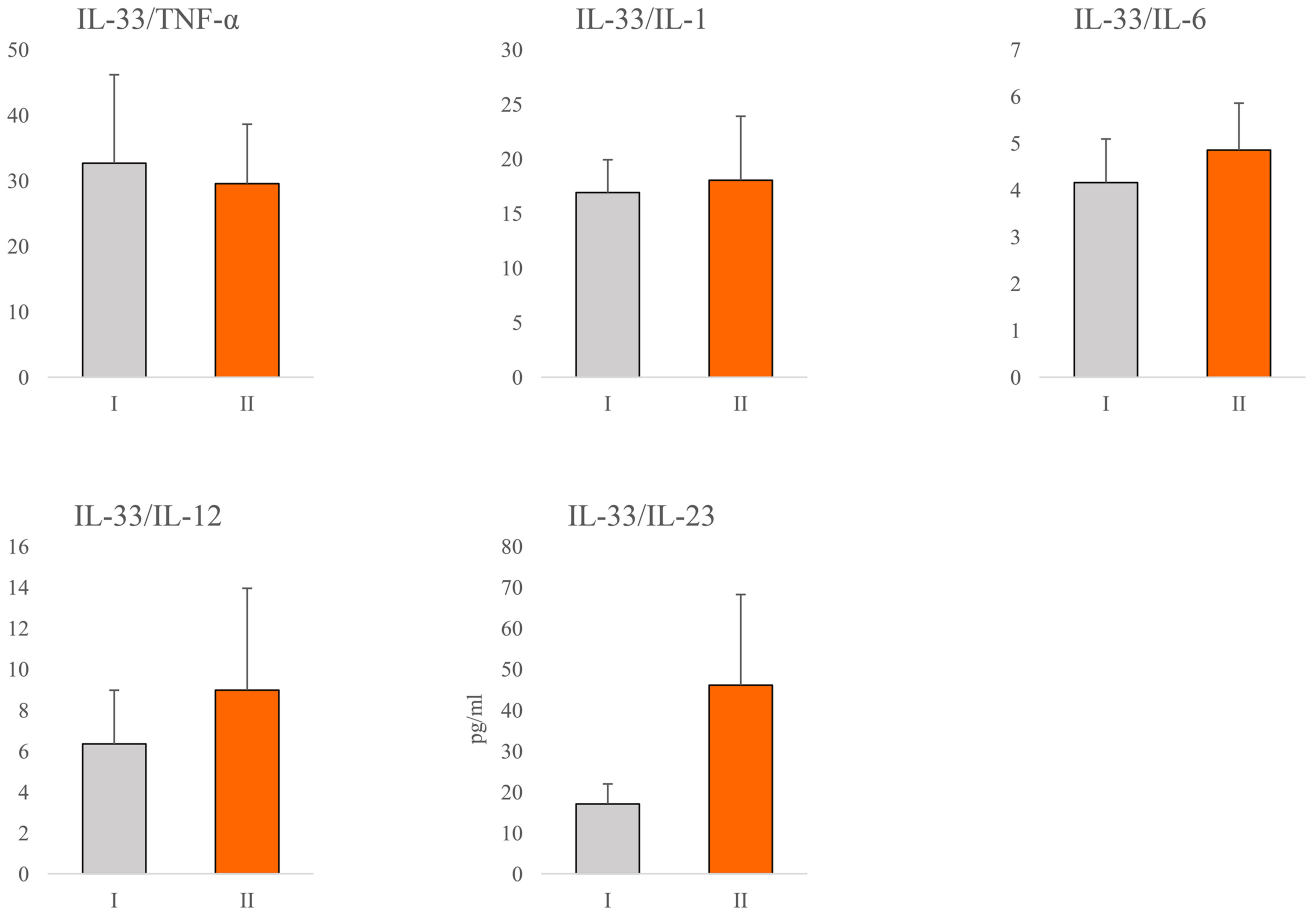
Ratio of IL-33 and proinflammatory cytokines. According to the disease severity, all COVID-19 patients were divided into two groups (I and II). The serum concentration of TNFα, IL-1β, IL-6, IL-12, IL-23, and IL-33 was determined by ELISA. The ratios of IL-33/TNF-α, IL-33/IL-1β, IL-33/IL-6, IL-33/IL-12, and IL-33/IL-23 were evaluated for each patient, separately. For statistical significance determination, Mann–Whitney Rank Sum test was used.

In order to determine the relationship between IL-33 and proinflammatory mediators in all stages of COVID-19, we analyzed the ratio of the systemic values of IL-33 with the values of TNF-α, IL-1β, IL-6, IL-12, and IL-23. We did not find a difference in the ratios between previously defined groups (Figure 2). Ratio IL-33/IL-23 was higher in group II, but this difference did not reach statistical significance (Figure 2).

Further, in patients with milder stage of COVID-19, positive correlation was detected between IL-33 and IL-1β (*p* = 0.024), IL-12 (*p* = 0.000), and IL-23 (*p* = 0.000), respectively. Analysis revealed a stronger positive correlation between the serum values of IL-33 and TNF-α (*p* = 0.000), IL-1β (*p* = 0.000), IL-6 (*p* = 0.000), IL-12 (*p* = 0.000), and IL-23 (*p* = 0.000) in COVID-19 patients with severe disease (Table 3). Moreover, analysis revealed a positive correlation between the serum values of IL-33 and IL-12 (*p* = 0.001), IL-33 and IL-6 (*p* = 0.001), and IL-6 and IL-12 (*p* = 0.001) in patients with COVID-19 (Supplementary Table 1).

**Table 3:** Correlation between interleukin (IL)-33 and proinflammatory cytokines.

	**IL-33**			
**Clin. stage**	**I**		**II**	
	**Pearson‘s rho**	***p* value**	**Pearson‘s rho**	***p* value**
**TNF-α**	0.029	0.751	0.605	0.000
**IL-1β**	0.201	0.024	0.399	0.000
**IL-6**	0.092	0.306	0.440	0.000
**IL-12**	0.313	0.000	0.565	0.000
**IL-23**	0.747	0.000	0.898	0.000

### IL-33 Correlates With Clinical Parameters of COVID-19

**Figure 3:**
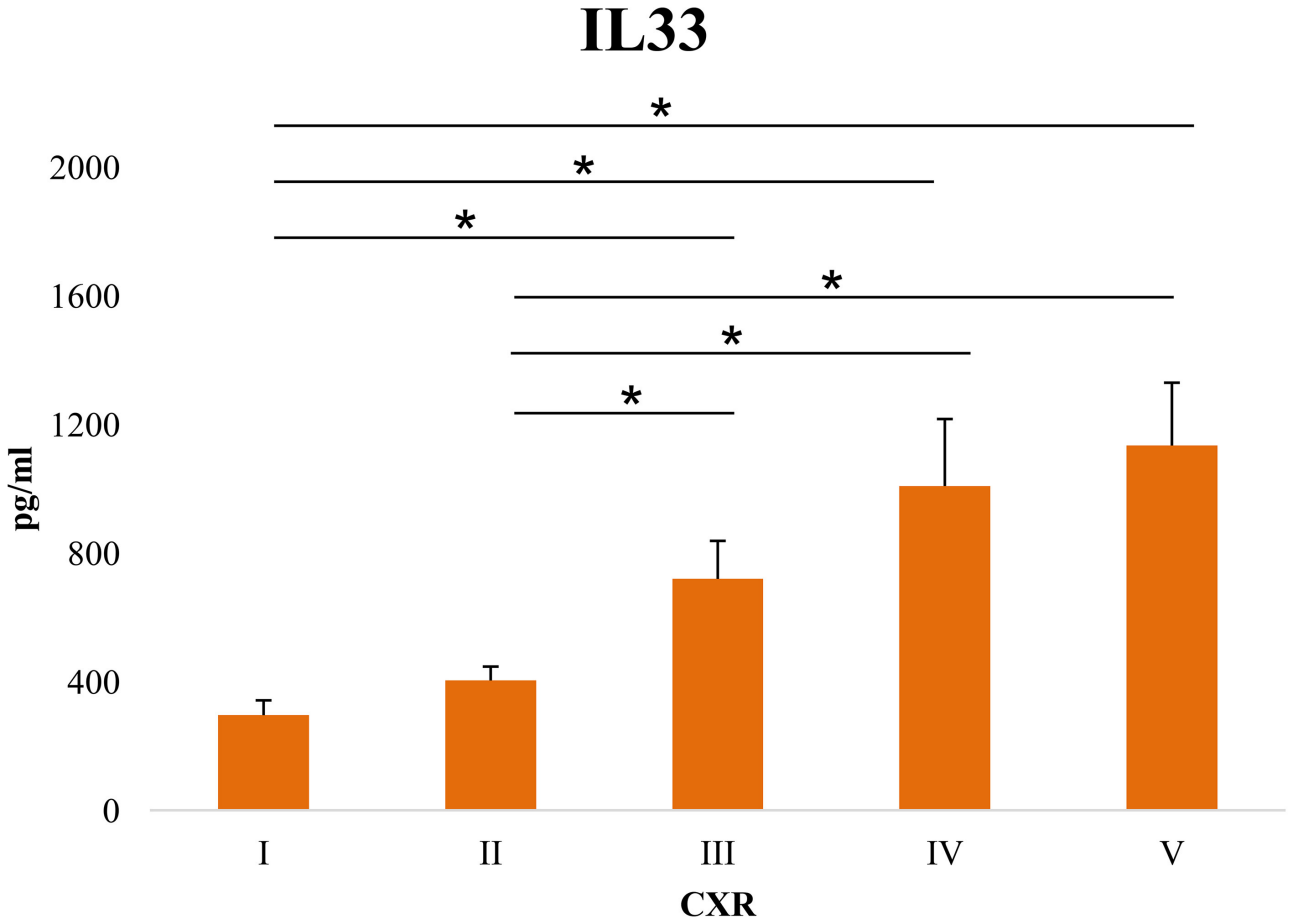
Level of serum IL-33 in patients sorted according to chest x-ray (CRX) findings. Using a digital portable anteroposterior (AP) technique, the chest X-ray findings were divided into five levels: (I) Normal finding (II) Interstitial thickening, {III) Focal consolidation, (IV) Multifocal consolidation (V) acute respiratory distress syndrome (ARDS). The serum concentration of IL-33 was determined by ELISA. For statistical significance determination, Mann–Whitney Rank Sum test was used. *P < 0.05.

Further, we have tested the correlation of the serum levels of proinflammatory cytokines with the clinical parameters of COVID-19. The results have shown a weak negative correlation between pO_2_ and serum IL-1β (*p* = 0.009), IL-12 (*p* = 0.018) and IL-33 (*p* = 0.018), and between SaO_2_ and serum IL-33 (*p* = 0.028) (Supplementary Table 2). The serum level of all cytokines of interest positively correlated with the CXR findings (Supplementary Table 2). The regression model for the independent variables, including the demographic features of the patients, clinical, biochemical parameters, and serum levels of innate immunity and dependent variable (COVID-19 severity) yielded significant results as shown in Supplementary Table 3. Male sex and advanced age are predictors of increased COVID-19 severity. Clinical findings such as dry cough, dyspnea, fatigue, and auscultation findings (attenuated breathing sound and crackles) are risk factors associated with developing severe COVID-19. When it comes to laboratory findings, lymphocyte and monocyte counts, levels of hemoglobin, and albumin were associated with decreased risk of disease progression. As expected, analyses of arterial blood gases were also associated with decreased risk of COVID-19 severity. Our results also illustrated that the serum levels of IL-1β, TNFα, IL-6, IL-12, IL-23, and IL-33 are risk factors associated with COVID-19 severity. The linear multiple regression model was calculated to predict the levels of IL-33 based on COVID-19 severity, IL-6, IL-1β, IL-12, TNF-α, and IL-23. A significant regression equation was established (Supplementary Table 4). The standardized beta values of COVID-19 severity (148.953, *p* = 0.049), IL-6 (−0.411, *p* = 0.003), IL-23 (0.896, *p* = 0.001), and IL-1β (0.858, *p* = 0.023) were significant, while beta values of IL-12 and TNF-α did not reach statistical significance (Supplementary Table 4). As shown in Figure 3, the serum values of IL-33 were significantly higher in patients with CXR score III, IV, and V compared with patients with CXR score I (*p* = 0.009; *p* = 0.002 and *p* = 0.001, respectively) and CXR score II (*p* = 0.01; *p* = 0.002, and *p* = 0.001, respectively). The ROC curve analysis of IL33 to differentiate mild from severe COVID-19 reviled that the optimum cut-off point for IL-33 was 332.08 pg/ml (AUC.634; Sensitivity 63.8%; Specificity 49.2%; 95% CI.562–0.705; *p* = 0.001).

## Discussion

In this study, we analyzed the relationship between clinical features, laboratory features, radiographic findings, and innate immunity cytokine profile (IL-33, TNF-α, IL-1β, IL-6, IL-12, and IL-23) with disease severity. Patients with COVID-19 were divided into two groups (mild/moderate and severe/critical) according to the criteria described in the material and methods. We found that gender and age correlate with disease severity. Most patients in the severe/critical group were male (81.8%) and older (over 64.5 years). This is in accordance with the study of Hu et al., where the authors suggested that age and gender may be risk factors for severe clinical outcomes (24). In our study, patients in the severe/critical group have a significantly higher frequency of dyspnea, fatigue, and auscultatory attenuated breathing sound and diffuse cracks in comparison to the mild/moderate group (Table 1). Also, the higher values of the neutrophil count, CRP, D dimer, PCT, LDH, urea, creatinine, CK, Ferritin, TBIL, AST, as well as lower values of lymphocyte and monocyte count, and albumin were detected in the group of patients with severe COVID-19 (Table 2). The same phenomenon was reported in the studies of Wang et al. (25) and Sánchez et al. (26). We detected higher values of white blood cell count, ALT, and lower values of red blood cells, platelets count, K^+^, and Na^+^ concentrations in patients with severe COVID-19, but the differences did not reach statistical significance (Table 2). Given the vast range of immune, biochemical, and metabolic disruptions during severe COVID-19, multiple organ failure is inevitable. As far as it is known, the thrombosis of various organs, renal, hepatic, cardiac, and neurological injury occurs more frequently as the severity of COVID-19 increases (27–29). In addition, our study revealed that PCT sera levels were significantly higher in the severe/critical group in comparison to the mild/moderate group. Since PCT level is not elevated in viral infections *per se*, this finding might be a marker of amplified systemic inflammatory response (e.g., cytokine storm) or secondary bacterial or fungal infections (27, 30).

Further on, we analyzed the CXR findings of 220 patients divided in the mild/moderate and severe/critical groups. As mentioned before, CXR findings are divided into five groups, where CXR I, II, III are expected findings in the mild/moderate group, and CXR IV and V are expected to be found in severe/critical forms of the disease. Radiographic findings such as multifocal consolidation and diffuse alveolar damage, denoted as CXR IV and V, respectively, are significantly more frequent in severe forms of COVID-19 that require supportive modes of ventilation, such as high-frequency ventilation (HFV), non-invasive (NIV), or invasive mechanical ventilation.

It is believed that immunopathology is the main mechanism in the genesis and progression of COVID-19 (12, 14). The cellular composition of the lung infiltrates in patients with COVID-19 pneumonia changes with the progression of the disease. Infiltrates in patients with moderate pneumonia include mainly lymphoid and dendritic cells, while the severe form of the disease is characterized by massive infiltration of macrophages and neutrophils (31, 32). In line with this finding, our results revealed an increment in neutrophil count in patients with severe COVID-19 (Table 2). Interestingly, we detected a decrement in monocyte count in the same patients (Table 2), which we believe may be due to the accumulation of these cells in the lungs and differentiation into macrophages. The destruction of the alveolar epithelium, which is directly related to the severity of the disease, is due to an intense immune response (33, 34). There is ample evidence that both, innate and acquired immunity participate in the immunopathogenesis of COVID-19 (12, 14, 35). However, the main pro-inflammatory mediators that play a key role in tissue destruction belong to the innate immune response (36, 37). In order to clarify the correlation between COVID-19 and innate immunity cytokines of interest, we analyzed serum cytokines in patients with COVID-19. The increased systemic values of TNF-α, IL-1β, IL-6, IL-12, IL-23, and IL-33 (Figure 1) support the prevalence of innate pro-inflammatory mediators in the serum of subjects with a severe form of COVID-19. The obtained results are in line with similar studies, which confirm the strong correlation between disease severity and increased values of some innate immunity-associated proinflammatory cytokines (36–39).

Interleukin-33 is a member of the IL-1 cytokine family that is expressed in barrier tissues and exerts pleiotropic functions. Its role in immune response regulation after cellular stress or damage is crucial and is involved in the pathogenesis of COVID-19 (40). The analysis of the published scRNAseq data of bronchoalveolar lavage fluid (BALF) from patients with mild to severe COVID-19 revealed a population of IL-33-producing cells that increases with the disease. These findings show that IL-33 production is linked to SARS-CoV-2 infection (41). Our result revealed a significantly higher systemic IL-33 concentration in patients with severe COVID-19, positive association between IL-33 and innate immunity mediators, and clinical parameters of COVID-19 (Figure 1, Table 3, Supplementary Table 2), suggesting the important role of this cytokine in the progression of COVID-19. In line with these are the results of other studies, which showed that disease severity is associated with an increased level of IL-33 (41–43).

The almost unchanged ratio between IL-33 and innate immunity mediators of interest during COVID-19 progression (Figure 2) indicates the stable dynamics of cytokine growth. Our results revealed a stronger positive correlation between serum values of IL-33 and innate immunity mediators TNF-α, IL-1β, IL-6, IL-12, and IL-23 in the severe form of COVID-19 (Table 3). Advanced stages of COVID-19 are characterized by high circulating and pulmonary concentrations of innate immunity cytokines (33, 44, 45). Signaling downstream of IL-1 family receptors, including IL-1β and IL-33 receptors, can activate MyD88 and elicit inflammation *via* production of high amounts of IL-1β, and NF-κB-induced cytokines such as TNF-α, IL-6, IL-12, and IL-23 (38, 46). In line with this, we found a positive mutual relation between the serum values of IL-33 and IL-12, IL-33 and IL-6, and IL-6 and IL-12 (Supplementary Table 1). The revelation of the positive correlation between the systemic IL-33, IL-6, and IL-12 levels in serum of COVID-19 patients suggested the synergistic effect of these cytokines in the pathogenesis of COVID-19 disease.

Significantly, disease severity was associated with age, neutrophil count, glucose, urea, creatinine, BIL T, BIL D, AST, LDH, CK, D dimer, CRP, PCT, TNF-α, IL-1β, IL-6, IL-12, IL-23, and IL-33 (Tables 1, 2; Supplementary Table 2). Among all the analyzed innate immunity cytokines, the strongest correlation was between IL-33 and disease severity (Supplementary Table 2). Serum levels greater than 332.08 pg/ml of IL-33 are associated with increased severity of COVID-19, indicating that IL-33 could be used as a prognostic biomarker in patients with a diagnosis of COVID-19. Interestingly, when CXR findings are compared with the sera levels of IL-33, a significant increase of IL-33 was detected in patients with CXR III, IV, and V findings. Focal consolidation, denoted as CXR III, is a CXR sign of mild to moderate cases of COVID-19. However, having in mind that elevated IL-33 is a marker of severe COVID-19 (19, 40), our result might imply that focal consolidation, alongside elevated IL-33 in the serum of patients might predict the progression of mild/moderate to severe/critical forms of the disease (Figure 3). As it is known, inflammation is closely related to the severity of COVID-19 (37). Increased inflammation consequently leads to more extensive tissue damage, and increased externalization of heat-shock proteins and alarmins. As IL-33 functions as both, alarmin and cytokine, it is possible that at first, as an alarmin, IL-33 facilitates inflammation, and then perpetuates it *via* interaction with other innate immunity cytokines or other immune cells, forming a circulus vitiosus of inflammation in the lungs.

Taking into account the results of this and similar studies, we believe that SARS-CoV-2 infection triggers IL-33 release from damaged epithelial cells in the lung (47). Released IL-33 initiates type 2 immune response that further leads to impaired antiviral activity and dysregulation of neutrophils (18). Dampened antiviral immunity results in delayed viral clearance and COVID-19 progression. In a more progressive stage of the disease, extensive injury of alveolar epithelial cells caused by strong interactions between the airway epithelium and activated immune cells is followed by overproduction of IL-33 (47). Under these circumstances, increased IL-33 facilitates lung inflammation by inducing the production of various innate proinflammatory cytokines (IL-1β, IL-6, TNF-α, IL-12, IL-23) in several target cells leading to the most severe forms of the disease (20, 48). Keeping in mind obtained data that IL-33 portrays an important role during severe form of COVID-19 followed by augmented inflammation, IL-33 might act as a promising therapeutic target which hinders COVID-19 severity.

### The Limitations and Strengths of the Study

The first limitation of the study is its cross-sectional design. Additional limitation includes a small sample size and incomplete epidemiological and clinical data follow-up. Due to the absence of a unified national platform for COVID-19 data consolidation, we only used confirmed cases from the University Clinical Center of Kragujevac (Covid Center). Due to the excluding criteria, there is a possibility of selection bias, as the third limitation of our study. Future research should include the analyzes of the linkage of variables using longitudinal study design.

The strength of our study is that it provides a detailed discussion regarding the pathophysiology of inflammatory markers in COVID-19. Well-defined inclusion criteria, data collection, and statistical interpretation were done.

## Data Availability Statement

The raw data supporting the conclusions of this article will be made available by the authors, without undue reservation.

## Ethics Statement

The study involving human participants was reviewed and approved by Ethics Committees of the University Clinical Center of Kragujevac, Serbia (Approval Number 01/20-406) Faculty of Medical Sciences, University of Kragujevac, Serbia (Approval Number 01-6776). The patients/participants provided their written informed consent to participate in this study.

## Author Contributions

SM and IJ: conceptualization. SM, NG, and MilenaJ: methodology. MilenaJ: software and formal analysis. IJ and VM: validation. SM, MarinaJ (6th author), NG, and MilenaJ: investigation. NA: resources. SL, NZ, and MiodragJ: data curation. SM: writing—original draft preparation. IJ and MarinaJ (2nd author): writing—review and editing. VV, MilanJ, and JS: visualization. IJ: supervision. IJ and NA: project administration. NA and ZM: funding acquisition. All authors have read and agreed to the published version of the manuscript.

## Funding

This work was supported by a grant from the Science Fund of the Republic of Serbia (CIBIRDS), Serbian Ministry of Education, Science and Technological Development (175069), project with PR China (06/2018).

## Conflict of Interest

The authors declare that the research was conducted in the absence of any commercial or financial relationships that could be construed as a potential conflict of interest.

## Publisher's Note

All claims expressed in this article are solely those of the authors and do not necessarily represent those of their affiliated organizations, or those of the publisher, the editors and the reviewers. Any product that may be evaluated in this article, or claim that may be made by its manufacturer, is not guaranteed or endorsed by the publisher.
