# Supplementary material for: IL 33 Correlates With COVID-19 Severity, Radiographic and Clinical Finding
Source: Front Med (Lausanne). 2021 Nov 30;8:749569. doi: 10.3389/fmed.2021.749569 (PMC8669591; doi:10.3389/fmed.2021.749569)
Supplement: Supplementary file 1 [file Data_Sheet_1.docx]

**Supplementary data**

**Supplementary table 1.** Correlation between proinflammatory cytokines in COVID-19 patients

|  | IL-12 | | IL-6 | |
| --- | --- | --- | --- | --- |
|  | Pearson`s rho | p value | Pearson`s rho | p value |
| IL-33 | 0.531 | 0.001 | 0.405 | 0.001 |
| IL-6 | 0.622 | 0.001 |  | |

**Supplementary table 2.** Correlation between proinflammatory cytokines and clinical parameters of COVID-19

|  | **pO_2_** | | **saturation** | | **CXR** | |
| --- | --- | --- | --- | --- | --- | --- |
|  | Pearson`s rho | p value | Pearson`s rho | p value | Pearson`s rho | p value |
| **TNF-α** | -0.011 | 0.870 | -0.003 | 0.970 | 0.187 | 0.004 |
| **IL-1β** | -0.170 | 0.009 | -0.098 | 0.136 | 0.304 | 0.000 |
| **IL-6** | -0.114 | 0.082 | -0.055 | 0.403 | 0.348 | 0.000 |
| **IL-12** | -0.155 | 0.018 | -0.087 | 0.185 | 0.311 | 0.000 |
| **IL-23** | -0.083 | 0.206 | -0.083 | 0.211 | 0.191 | 0.003 |
| **IL-33** | -0.156 | 0.018 | -0.147 | 0.028 | 0.304 | 0.000 |

**Supplementary table 3.** Linear regression analysis for dependent variable (COVID-19 severity)

|  | **Unstandardized coefficients (*B*)** | **Std. error** | **Standardized coefficients (beta)** | ***t*** | ***p*** |
| --- | --- | --- | --- | --- | --- |
| **Age** | 0.008 | 0.002 | 0.247 | 4.172 | 0.001 |
| **Sex (Male)** | 0.160 | 0.069 | 0.140 | 2.322 | 0.021 |
| **Clinical manifestations** | | | | | |
| Dry cough | 0.127 | 0.063 | 0.121 | 2.004 | 0.046 |
| Dyspnea | 0.183 | 0.061 | 0.180 | 2.984 | 0.003 |
| Fatigue | 0.183 | 0.063 | 0.176 | 2.906 | 0.004 |
| **Auscultatory findings** | | | | | |
| Attenuated breathing sound | 0.145 | 0.061 | 0.144 | 2.386 | 0.018 |
| Crackles | 0.210 | 0.060 | 0.211 | 3.532 | 0.001 |
| **Blood routine examination** | | | | | |
| Neutrophil | 0.008 | 0.002 | 0.223 | 3.703 | 0.001 |
| Lymphocyte | -0.010 | 0.003 | -0.228 | -3.770 | 0.001 |
| Monocyte | -0.026 | 0.006 | -0.28 | -4.709 | 0.001 |
| Hemoglobin | -0.004 | 0.002 | -0.145 | -2.406 | 0.017 |
| **Biochemical examination** | | | | | |
| Glucose | 0.023 | 0.008 | 0.167 | 2.764 | 0.006 |
| Urea | 0.029 | 0.006 | 0.305 | 5.250 | 0.001 |
| Creatinin | 0.001 | 0.000 | 0.162 | 2.697 | 0.007 |
| BILT | 0.033 | 0.014 | 0.274 | 2.413 | 0.018 |
| BILD | 0.090 | 0.037 | 0.295 | 2.434 | 0.018 |
| AST | 0.002 | 0.001 | 0.171 | 2.811 | 0.005 |
| Albumin | -0.019 | 0.005 | -0.227 | -3.791 | 0.001 |
| LDH | 0.001 | 0.00008 | 0.416 | 6.933 | 0.001 |
| CK | 0.0002 | 0.0001 | 0.160 | 2.601 | 0.010 |
| CRP | 0.001 | 0.0003 | 0.257 | 4.369 | 0.001 |
| PCT | 0.161 | 0.035 | 0.271 | 4.623 | 0.001 |
| Feritin | 0.0001 | 0.00003 | 0.242 | 3.794 | 0.0001 |
| **Arterial blood gases** | | | | | |
| p0_2_ | -0.131 | 0.012 | -0.571 | -11.403 | 0.001 |
| Sa0_2_ | -0.036 | 0.004 | -0.497 | -9.379 | 0.001 |
| pH | -1.337 | 0.618 | -0.132 | -2.164 | 0.031 |
| **Coagulation function** | | | | | |
| D dimer | 0.039 | 0.010 | 0.222 | 3.735 | 0.001 |
| **Cytokines** | | | | | |
| IL-1β | 0.001 | 0.0002 | 0.210 | 3.256 | 0.001 |
| TNF-α | 0.000328 | 0.000125 | 0.170 | 2.619 | 0.009 |
| IL-6 | 0.000279 | 0.00008 | 0.204 | 3.163 | 0.002 |
| IL-12 | 0.0003 | 0.00010 | 0.198 | 3.064 | 0.002 |
| IL-23 | 0.00007 | 0.00002 | 0.178 | 2.737 | 0.007 |
| IL-33 | 0.00009 | 0.00002 | 0.222 | 3.418 | 0.001 |

**Supplementary table 4**. Coefficients and R^2^ for the regression model; dependent variable IL33

|  | **Unstandardized coefficients (*B*)** | **Std. error** | **Standardized coefficients (beta)** | ***t*** | ***p*** |
| --- | --- | --- | --- | --- | --- |
| Constant | 148.876 | 60.322 |  | 2.468 | 0.014 |
| COVID-19 severity | 148.953 | 76.473 | 0.065 | 1.948 | 0.049 |
| IL-6 | -0.411 | 0.139 | -0.131 | -2.960 | 0.003 |
| IL-1β | 0.858 | 0.374 | 0.093 | 2.296 | 0.023 |
| IL-23 | 0.869 | 0.044 | 0.869 | 19.831 | 0.001 |
| IL-12 | -0.001 | 0.196 | 0.000 | -0.005 | 0.996 |
| TNF-α | 0.171 | 0.206 | 0.039 | 0.831 | 0.407 |
| R^2^ | 0.771 |  |  |  |  |
| Adj. R^2^ | 0.765 |  |  |  |  |
| F (df=6) | 123.36 |  |  |  | 0.001 |
